# Physical activity and HRQoL among adolescents: the mediating roles of cognitive reappraisal and stress self-management

**DOI:** 10.3389/fpubh.2026.1914300

**Published:** 2026-09-11

**Authors:** Chuchu Li, Shuxin Zhang, Huilin Wang, Ziqing Xu

**Affiliations:** 1 Moray House School of Education and Sport, The University of Edinburgh, Edinburgh, United Kingdom; 2 Chinese International College, Dhurakij Pundit University, Bangkok, Thailand; 3 School of Business, Hunan University of Science and Technology, Xiangtan, China; 4 Business School, Guangdong Ocean University, Yangjiang, China

**Keywords:** adolescents, cognitive reappraisal, HRQoL, physical activity, stress self-management

## Abstract

**Introduction:**

Physical activity is important for adolescent health, but the psychological pathways linking physical activity to health-related quality of life (HRQoL) remain unclear. This study examined the association between physical activity and HRQoL among secondary school students, focusing on the mediating roles of cognitive reappraisal and stress self-management.

**Methods:**

A paper-based questionnaire survey was conducted in April 2026 among secondary school students in Jiangsu Province, China. A total of 500 questionnaires were distributed, and 420 valid responses were retained. Physical activity, cognitive reappraisal, stress self-management, and HRQoL were measured using validated scales. SPSS 26.0 and AMOS 23.0 were used to conduct descriptive statistics, reliability and validity tests, correlation analysis, and structural equation modeling.

**Results:**

The structural model showed a good fit to the data. Physical activity was positively associated with cognitive reappraisal and stress self-management. Cognitive reappraisal was positively associated with stress self-management, and both cognitive reappraisal and stress self-management were positively associated with HRQoL. The mediation results were consistent with a significant indirect association between physical activity and HRQoL through cognitive reappraisal and stress self-management.

**Discussion:**

Physical activity was associated with better HRQoL among adolescents, partly through cognitive reappraisal and stress self-management. These findings suggest that school-based physical activity may support adolescents' quality of life by strengthening psychological regulation and stress management.

## 1 Introduction

Adolescence is a critical developmental stage characterized by profound physical, emotional, and social transitions that shape individuals' long-term health and wellbeing (1). During this period, health-related quality of life (HRQoL), a multidimensional construct encompassing physical, psychological, social, and daily functioning—has become an increasingly important indicator for assessing adolescent development and wellbeing (2, 3). In recent years, adolescent mental health has emerged as a major global public health concern. According to the WHO (4), approximately one in seven adolescents aged 10–19 years is affected by a mental disorder, accounting for a substantial proportion of the global burden of disease in this age group. Anxiety and depressive disorders are particularly prevalent, affecting millions of adolescents worldwide and raising growing concerns about their long-term developmental outcomes.

In the Chinese context, similar challenges have been widely reported. National-level evidence suggests that approximately 20.3% of Chinese adolescents experience psychological symptoms, reflecting a considerable mental health burden within this population (5). At the behavioral level, physical inactivity remains highly prevalent, with nearly half of adolescents failing to achieve even 30 min of moderate-to-vigorous physical activity per day, and only a small proportion meeting the World Health Organization's recommendation of at least 60 min daily. In addition, academic pressure has been consistently identified as a major contextual stressor contributing to elevated levels of anxiety and depression among Chinese adolescents (6). Taken together, these findings suggest that Chinese adolescents are exposed to a combination of high academic demands and insufficient physical activity, both of which may negatively influence their psychological wellbeing and overall quality of life.

A substantial body of literature has consistently demonstrated the beneficial effects of physical activity on adolescent mental health and wellbeing. Multiple studies have shown that higher levels of physical activity are associated with lower depressive symptoms (7), reduced anxiety (8), lower perceived stress (9), and improved HRQoL (10) across different cultural contexts. These findings have established physical activity as a robust behavioral predictor of adolescent psychological and health outcomes. In parallel, research grounded in the Process Model of Emotion Regulation has highlighted cognitive reappraisal as one of the most adaptive emotion regulation strategies for enhancing psychological wellbeing (11). Empirical and meta-analytic evidence indicated that individuals who more frequently engage in cognitive reappraisal tend to experience better psychological adjustment (12) and lower levels of depressive and anxiety symptoms across adolescent and adult populations (13). However, this literature has primarily focused on intra-individual emotional processes and has paid limited attention to behavioral antecedents such as physical activity. Similarly, research based on Stress and Coping Theory has extensively examined stress self-management and coping strategies as critical determinants of adolescents' psychological adaptation and quality of life (14, 15). A large body of empirical studies and systematic reviews has confirmed that adaptive coping strategies are associated with better mental health outcomes and higher quality of life (16–18). Nevertheless, this stream of research has largely developed independently of emotion regulation frameworks and has rarely been integrated with behavioral determinants such as physical activity, resulting in a fragmented understanding of how behavioral engagement, cognitive regulation, and coping processes jointly contribute to adolescent wellbeing and HRQoL.

Despite the substantial body of evidence supporting the relationships among physical activity, emotion regulation, coping processes, and adolescent wellbeing, the existing literature remains fragmented in several important respects. First, previous studies have largely examined the associations between physical activity and psychological outcomes, including cognitive and emotional functioning, without systematically investigating how different psychological processes operate together to influence HRQoL. Second, although research has shown that physical activity may be associated with adaptive emotion regulation capacities and stress-related coping resources, these relationships have typically been examined in separate lines of inquiry. As a result, limited attention has been given to the potential interconnections among behavioral engagement, cognitive regulation, and stress management processes. Third, while emotion regulation and coping research have both identified important predictors of adolescent adjustment and wellbeing, few studies have integrated these perspectives to examine how cognitive regulation may facilitate subsequent stress management processes and ultimately contribute to HRQoL. Consequently, the mechanisms through which physical activity may be translated into improved HRQoL through interconnected psychological resource processes remain insufficiently understood.

In response to these research gaps, the present study develops and tests an integrated model linking physical activity, cognitive reappraisal, stress self-management, and HRQoL among adolescents. Using survey data collected from secondary school students in Jiangsu Province, China, this study examines whether cognitive reappraisal and stress self-management serve as psychological mechanisms through which physical activity is associated with HRQoL. Specifically, drawing on Conservation of Resources theory, we investigate both the mediating roles of these two resource-related processes in explaining the relationship between physical activity and adolescents' wellbeing. This study contributes to the existing literature in several ways. First, it extends current research on physical activity and adolescent HRQoL by moving beyond direct-effect explanations and examining the psychological processes that may underlie this relationship. Second, by integrating cognitive reappraisal and stress self-management within a unified resource-based framework, this study provides a more comprehensive understanding of how behavioral resources may be transformed into psychological and wellbeing outcomes. Finally, given the in-creasing mental health challenges faced by adolescents in academically demanding environments, the findings may offer practical implications for schools, educators, and health practitioners seeking to promote adolescent wellbeing through both physical activity interventions and psychological resource development.

## 2 Literature review and hypothesis development

### 2.1 Conservation of resources theory

The Conservation of Resources theory, originally developed by Hobfoll (19), provides a comprehensive theoretical framework for understanding how individuals obtain, retain, and protect valued resources in the context of environmental demands and stressors. Unlike traditional stress models that primarily emphasize external stressors, Conservation of Resources theory posits that psychological stress occurs when individuals experience actual resource loss, anticipate potential resource loss, or fail to achieve sufficient resource gain following resource investment (20).

A central assumption of Conservation of Resources theory is that resources are not static but operate in dynamic and cumulative processes. Individuals who possess greater initial resources are more likely to acquire additional resources over time, leading to resource gain spirals, whereas those who experience initial resource depletion are more vulnerable to further losses, resulting in loss spirals (21). In this sense, resources are understood as entities that tend to aggregate and interact over time, shaping individuals' adaptive capacity and psychological functioning. Empirical and conceptual extensions of Conservation of Resources theory further emphasize that resources may include personal characteristics, conditions, energies, and contextual supports, which jointly influence individuals' ability to cope with stress and maintain wellbeing (22, 23).

Importantly, Conservation of Resources theory has received substantial empirical support across a wide range of psychological and organizational contexts. A large body of research has applied Conservation of Resources theory to explain stress, burnout, work engagement, and health-related outcomes, consistently demonstrating that resource loss is strongly associated with negative psychological states, whereas resource gain is linked to improved wellbeing (22, 24, 25). Previous studies have further confirmed that resource-based processes play a central role in predicting both psychological strain and positive functioning across diverse populations (26, 27). These empirical findings provide strong support for the core assumptions of Conservation of Resources theory and highlight its applicability in explaining individual differences in coping and wellbeing outcomes.

From this perspective, Conservation of Resources theory provides a useful lens for understanding adolescent development, particularly in contexts characterized by sustained academic pressure and psychosocial demands. Adolescents must continuously allocate cognitive, emotional, and physical resources to manage school-related requirements, social expectations, and developmental challenges. In the present study, however, physical activity is not conceptualized as an energy resource in Hobfoll's original sense. Rather, it is understood as a resource-investment behavior because participation requires adolescents to invest time, effort, and physical energy. Regular engagement in physical activity may, in turn, be associated with the maintenance, recovery, or development of valued resources, including physical fitness, vitality, self-regulatory capacity, and adaptive coping skills. Previous research has similarly applied Conservation of Resources theory to explain physical activity participation and the psychological resource gains associated with physical activity (28). Cognitive reappraisal and stress self-management are therefore positioned as personal regulatory resources within this broader resource-gain process. Examining the associations among physical activity, these regulatory resources, and HRQoL allows the proposed model to be grounded in Conservation of Resources theory without conflating physical activity with physical energy.

### 2.2 Physical activity, cognitive reappraisal and stress self-management

Previous research has linked physical activity among adolescents to better cognitive functioning, more positive emotional experiences, and stronger self-regulatory capacities (29). These findings suggest that physical activity may provide a behavioral context that supports adolescents' ability to respond adaptively to academic and psychosocial demands. One particularly relevant regulatory process is cognitive reappraisal, which refers to changing the interpretation of an emotion-eliciting or stressful situation in order to modify its emotional impact (30, 31). As an antecedent-focused emotion regulation strategy, cognitive reappraisal occurs before an emotional response is fully developed and allows individuals to reconsider the meaning of a situation in a less threatening or more constructive way (32). For adolescents facing academic and social pressures, the use of cognitive reappraisal may therefore be particularly important for maintaining emotional stability and psychological adjustment.

Although direct research on the relationship between physical activity and cognitive reappraisal remains relatively limited, several empirical studies have provided support for a positive association. Giles et al. (33) found that habitual exercise was associated with greater success in a laboratory-based cognitive reappraisal task. Wu et al. (34) further found that physical activity was positively associated with cognitive reappraisal among young adults, with cortical thickness in the right rostral anterior cingulate cortex partially accounting for this association. More recently, Chen et al. (35) identified a significant positive association between physical activity and cognitive reappraisal among junior secondary school students. Beyond these direct associations, research on executive functioning provides a possible explanation for why physical activity may be related to cognitive reappraisal. Reappraising an emotional situation requires individuals to disengage from an initial interpretation, consider alternative meanings, inhibit dominant emotional responses, and retain relevant information during the regulation process. Physical activity has been positively associated with cognitive flexibility (36, 37), inhibitory control (38, 39), and working memory (40, 41). Neuropsychological research has also linked physical activity to the structure and functioning of prefrontal and anterior cingulate regions involved in cognitive control and emotion regulation (42–44). Although these studies do not directly demonstrate that physical activity improves cognitive reappraisal, they identify cognitive capacities and neural systems that may help explain the positive associations reported in the direct empirical studies. Therefore, adolescents with higher levels of physical activity may be more likely to use cognitive reappraisal when responding to emotional and stressful experiences.

Based on the literature, this study proposes the following hypotheses:

**Hypothesis 1 (H1):** *Physical activity has a positive and significant association with cognitive reappraisal*.

Stress self-management is conceptually distinct from perceived stress, psychological distress, resilience, and general coping (45). Whereas perceived stress reflects the extent to which individuals experience situations as stressful, stress self-management refers to the regular use of self-initiated behaviors intended to manage stress and support recovery. In the present study, these behaviors include the use of stress-control methods, adequate sleep, relaxation, meditation, constructive thoughts before sleep, and appropriate pacing to prevent excessive tiredness.

A broader body of evidence suggests that physical activity is associated with more favorable stress-related experiences and recovery processes. VanKim and Nelson (46) found that vigorous physical activity was associated with lower perceived stress among university students. Similarly, Teuber et al. (47), using a longitudinal daily-level design, reported that leisure-time physical activity was associated with stress load and recovery experiences among university students. Although these studies did not directly measure stress self-management, they suggest that physical activity may provide an important behavioral context for regulating stress and facilitating recovery.

More direct evidence has linked physical activity to the use of stress-management behaviors. Vogel et al. (48) found that physically active individuals were more likely to use active stress-management strategies, including outdoor and indoor physical activity, yoga, meditation, and gardening, and were less likely to rely on potentially maladaptive responses such as excessive sleeping or eating. More importantly, Zhang et al. (15) directly examined physical activity and stress self-management among Chinese middle school students. Using the Health-Promoting Lifestyle Profile to assess these constructs as separate behavioral dimensions, they found a significant positive association between physical activity and stress self-management. These findings suggest that adolescents with higher levels of physical activity may also engage more frequently in health-promoting behaviors aimed at managing and recovering from stress.

Based on the literature, this study proposes the following hypotheses:

**Hypothesis 2 (H2):** *Physical activity has a positive and significant association with stress self-management*.

Research in emotion regulation theory has consistently identified cognitive reappraisal as a key adaptive strategy for managing emotional responses to stress. According to the Process Model of Emotion Regulation, cognitive reappraisal operates at an early stage of emotional processing and allows individuals to reinterpret stressors in less threatening ways, thereby reducing emotional reactivity (32).

Empirical studies have shown that individuals who frequently use cognitive reappraisal tend to experience lower levels of stress and demonstrate more adaptive coping strategies (49). Meta-analytic evidence further confirms that cognitive reappraisal is positively associated with adaptive coping and negatively associated with maladaptive stress responses (50, 51).

Based on the literature, this study proposes the following hypotheses:

**Hypothesis 3 (H3):** *Cognitive reappraisal has a positive and significant association with stress self-management*.

### 2.3 Cognitive reappraisal, stress self-management and HRQoL

Cognitive reappraisal is defined as a deliberate cognitive strategy in which individuals reinterpret potentially emotion-eliciting situations to alter their emotional impact (52). Empirical evidence indicates that cognitive reappraisal is associated with lower levels of negative affect, reduced perceived stress, and better psychological wellbeing across both adult and adolescent populations (53–55). In adolescence, frequent use of cognitive reappraisal has been linked to improved psychosocial functioning and decreased internalizing symptoms such as anxiety and depression, suggesting that it represents a key adaptive cognitive resource that may support broader aspects of mental health (56, 57). HRQoL encompasses multiple domains including physical, psychological, social, and daily functioning (58). Given that cognitive reappraisal contributes to positive psychological adjustment and emotion regulation, it is theoretically plausible that adolescents who engage in more frequent cognitive reappraisal will experience higher HRQoL.

Based on the literature, this study proposes the following hypotheses:

**Hypothesis 4 (H4):** *Cognitive reappraisal has a positive and significant association with HRQoL*.

Stress self-management refers to the regular use of self-initiated behaviors intended to regulate and recover from stress in daily life. These behaviors may include the use of stress-control methods, adequate sleep, relaxation, meditation, and appropriate pacing to prevent excessive tiredness. Prior research has consistently shown that effective stress management is associated with reduced psychological distress, higher life satisfaction, and improved overall functioning (59–61). Although these studies have primarily focused on psychological adjustment and emotional wellbeing, HRQoL represents a broader construct that integrates psychological, social, and physical dimensions of functioning. It is therefore reasonable to infer that adolescents who exhibit stronger stress self-management skills may also report higher HRQoL, particularly when coping with the academic and social demands characteristic of this developmental stage.

Based on the literature, this study proposes the following hypotheses:

**Hypothesis 5 (H5):** *Stress self-management has a positive and significant association with HRQoL*.

### 2.4 Mediation effects

Physical activity has been widely established as a behavioral factor that contributes to adolescents' HRQoL. Meta-analyses and large-scale empirical studies have consistently shown that higher levels of physical activity are associated with better physical functioning (62), psychological wellbeing (63), and social adaptation (64). Despite these robust associations, the psychological mechanisms through which physical activity may be linked to improved HRQoL remain underexplored.

Existing research provides varying levels of support for the individual components of the proposed model. Studies have shown that adolescents who are more physically active tend to exhibit better cognitive functioning and emotion-regulation-related capacities. More direct empirical research has also reported positive associations between physical activity and cognitive reappraisal (33–35, 49). Evidence concerning stress self-management is comparatively limited. Much of the previous literature has examined perceived stress, psychological distress, coping, resilience, or stress recovery rather than the specific self-initiated stress-management behaviors assessed in the present study. Nevertheless, direct research among adolescents has indicated that physical activity is positively associated with stress self-management behaviors (15). These findings suggest that engagement in physical activity may support the maintenance or development of internal regulatory resources that facilitate adaptive functioning, although the relevant processes have generally been investigated separately.

Research on emotion regulation and coping further indicates that adolescents capable of reinterpreting stressful events in more adaptive ways are more likely to engage in productive coping strategies and maintain better psychological adjustment (65–68). However, these studies primarily concern general coping and psychological adaptation and do not directly establish that cognitive reappraisal leads to the specific stress self-management behaviors measured in the present study. The proposed association between cognitive reappraisal and stress self-management should therefore be understood as theoretically informed and supported by evidence from closely related constructs, rather than as a firmly established relationship. Moreover, effective stress management has been linked to higher HRQoL, including improved psychological, social, and functional outcomes (69–71). Nevertheless, direct evidence involving the specific stress self-management construct adopted in this study also remains limited.

Although these relationships have generally been examined separately, existing evidence suggests that physical activity may be associated with HRQoL through a sequential process involving cognitive reappraisal and stress self-management. From the perspective of Conservation of Resources theory, physical activity can be understood as a resource-investment behavior that may support the development and maintenance of personal regulatory resources. Cognitive reappraisal enables adolescents to reinterpret stressful situations in less threatening ways and reduce unnecessary resource depletion, which may facilitate the use of stress self-management behaviors, such as relaxation, adequate sleep, meditation, stress-control methods, and appropriate pacing. These behaviors may further help adolescents conserve and restore personal resources, thereby contributing to better psychological, social, and functional wellbeing. Accordingly, cognitive reappraisal and stress self-management may operate sequentially in the relationship between physical activity and HRQoL.

Based on the literature, this study proposes the following hypotheses:

**Hypothesis 6 (H6):** *Cognitive reappraisal and stress self-management mediate the relationship between physical activity and HRQoL*.

**Figure 1:**
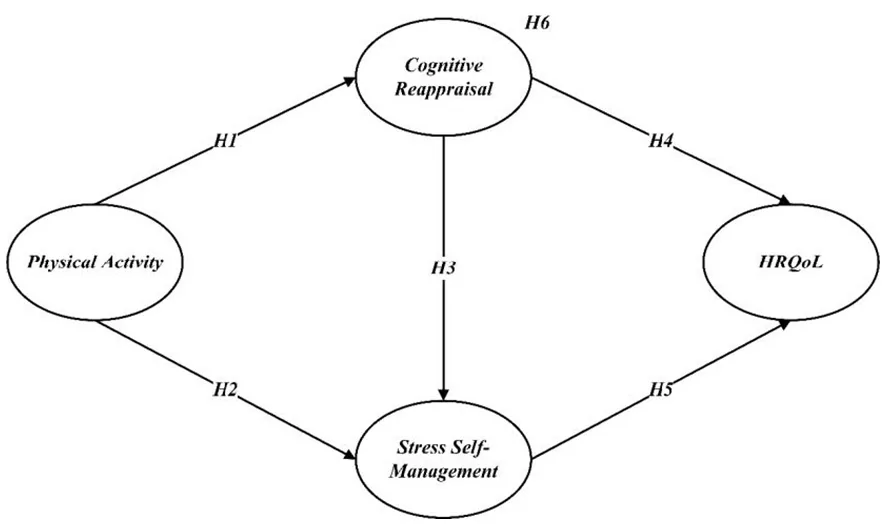
Hypothesis model.

All hypotheses are summarized in Figure 1.

## 3 Methodology

### 3.1 Participants and procedures

This study used a paper-based questionnaire survey and employed convenience and snowball sampling. The survey was conducted in April 2026 in two secondary schools in Jiangsu Province, China. Students were eligible to participate if they were enrolled in Grades 7–9 at one of the participating schools and were able to understand and complete the questionnaire independently. Snowball sampling was conducted at the class level: after the initial classes were contacted through convenience sampling, participating class teachers introduced the researchers to teachers of other eligible classes within the same schools, who then invited their students to participate. The researchers printed the questionnaires and distributed them to class teachers, who then passed them on to students in their classes. Before completing the questionnaire, all participants were informed of the purpose of the study and were told that their participation was voluntary and anonymous. Students completed the questionnaires independently, with support from researchers and teachers available if needed.

A total of 500 paper questionnaires were distributed. Questionnaires were excluded if they contained missing responses on the main study variables, showed highly repetitive response patterns, or were otherwise incomplete. No additional post-survey exclusion criteria were applied. After excluding invalid responses, such as questionnaires with missing values or highly repetitive response patterns, 420 valid questionnaires were retained, resulting in a valid response rate of 84.0%. The demographic characteristics of the participants are presented in Table 1. Overall, the sample showed a relatively balanced gender distribution, with 195 males (46.4%) and 225 females (53.6%). The participants were also relatively evenly distributed across Grade 7, Grade 8, and Grade 9, accounting for 34.8%, 34.0%, and 31.2% of the sample, respectively. Most students reported moderate to high levels of academic stress, suggesting that academic pressure was a common experience among the participants. In terms of sleep duration, the majority of students reported sleeping at least seven hours per night. In addition, participation in school sports clubs, sports teams, or extracurricular physical training was relatively balanced, with 46.7% reporting participation and 53.3% reporting non-participation.

**Table 1:** Demographic characteristics of participants (*N* = 420).

Profiles	Survey (%)
Gender	
Male	46.4
Female	53.6
Grade	
Grade 7	34.8
Grade 8	34.0
Grade 9	31.2
Perceived academic stress	
Low	9.5
Moderate	26.7
Relatively high	43.6
Very high	20.2
Sleep duration	
Less than 6 h	10.5
6–7 h	13.6
7–8 h	45.9
More than 8 h	31.0
Participation in school sports clubs, sports teams, or extracurricular physical training	
Yes	46.7
No	53.3

### 3.2 Instruments

All instruments were selected based on their established use and previously reported psychometric properties in relevant populations. For the reflective multi-item measures, reliability and validity were further examined in the present sample.

Physical activity was measured using the Physical Activity Rating Scale revised by Liang (72). This scale assesses students' physical activity levels from three aspects: exercise intensity, exercise duration, and exercise frequency. Each item was rated using five response options, with higher scores indicating a higher level of physical activity. A sample item is: “What is the intensity of physical activity that you usually participate in during the past month?” The total physical activity score was calculated using the formula: physical activity score = physical activity intensity score × (physical activity duration score – 1) × physical activity frequency score. Following the established PARS-3 scoring criteria, total scores of 19 or below were classified as low physical activity, scores from 20 to 42 as moderate physical activity, and scores of 43 or above as high physical activity. The PARS-3 was designed as a behavioral composite index in which exercise intensity, duration, and frequency represent distinct and non-interchangeable components that jointly determine the overall level of physical activity. Therefore, it was treated as an observed composite variable rather than a reflective latent construct. Accordingly, internal consistency coefficients and confirmatory factor analysis were not considered appropriate for this measure.

Cognitive reappraisal was measured using items adapted from the Emotion Regulation Questionnaire-Short Form developed by Preece et al. (73), which demonstrated satisfactory psychometric properties in its original validation study. The scale includes six items assessing how individuals regulate their emotions by changing the way they think about a situation. Participants responded on a five-point Likert scale ranging from 1 = strongly disagree to 5 = strongly agree. A sample item is: “When I want to feel more positive emotion (such as joy or amusement), I change the way I'm thinking about the situation.” Higher scores indicate a higher level of cognitive reappraisal.

Stress self-management was measured using items adapted from the stress management subscale of the Health-Promoting Lifestyle Profile (HPLP) developed by Pinar et al. (74), an instrument with previously established reliability and validity. This scale contains six items, which assess the frequency of stress management behaviors, including stress control, sleep, relaxation, bedtime thoughts, daily relaxation or meditation, and pacing to prevent tiredness. Participants responded on a five-point Likert scale ranging from 1 = never to 5 = always. A sample item is: “Use stress control methods.” Higher scores indicate better stress self-management.

HRQoL was measured using the KIDSCREEN-10 Index (58). This brief scale is designed to assess general HRQoL among children and adolescents and has demonstrated satisfactory psychometric properties across different adolescent populations. It includes 10 items covering physical wellbeing, psychological wellbeing, autonomy, parent relations, peer relations, and school-related functioning. Participants were asked to respond based on their experiences during the past week. Responses were rated on a five-point scale ranging from 1 = never to 5 = always. A sample item is: “Have you felt fit and well?” Higher scores indicate better HRQoL. Negatively worded items were reverse-coded before analysis.

### 3.3 Data analysis

Data analysis was conducted using SPSS 26.0 and AMOS 23.0. Descriptive statistics and correlation analyses were first performed to examine the basic characteristics of the variables and the associations among physical activity, cognitive reappraisal, stress self-management, and HRQoL. Reliability was assessed using Cronbach's alpha coefficients for the reflective multi-item measures, and confirmatory factor analysis (CFA) was conducted to examine the measurement model comprising cognitive reappraisal, stress self-management, and HRQoL. As PARS-3 is a composite index rather than a reflective scale, it was not included in the internal consistency or CFA analyses. To provide sample-specific evidence of its measurement performance, known-groups validity was examined by comparing PARS-3 scores between students who did and did not participate in school sports clubs, sports teams, or extracurricular physical training using an independent-samples *t*-test.

Structural equation modeling (SEM) was then used to test the hypothesized relationships among the study variables, including the direct association between physical activity and HRQoL and the indirect associations through cognitive reappraisal and stress self-management. Gender, grade, academic stress, sleep duration, and sports participation were included as control variables in the structural model. Model fit was evaluated using commonly recommended fit indices, including χ^2^/df, CFI, TLI, RMSEA, and SRMR. The significance of indirect effects was tested using the bootstrapping method with 5,000 resamples and 95% confidence intervals.

Because all variables were measured using self-reported questionnaires, common method variance (CMV) was examined using Harman's single-factor test. The results showed that the first unrotated factor accounted for 38.446% of the total variance, which was below the commonly used threshold of 40%. This suggests that CMV was not a serious concern in the present study.

## 4 Results

### 4.1 Measurement model

Before testing the structural model, the reliability and validity of the measurement model were examined. Physical activity was calculated using the established PARS-3 formula based on exercise intensity, duration, and frequency. Because these three components are distinct and non-interchangeable indicators that jointly form the physical activity score, PARS-3 was treated as an observed composite variable rather than a reflective latent construct. Therefore, internal consistency coefficients and confirmatory factor analysis were not considered appropriate for this measure. The measurement model included three latent constructs: cognitive reappraisal, stress self-management, and HRQoL.

As shown in Table 2, all standardized factor loadings were above the recommended threshold of 0.70. Specifically, the factor loadings ranged from 0.758 to 0.778 for cognitive reappraisal, from 0.740 to 0.802 for stress self-management, and from 0.734 to 0.779 for HRQoL. These results indicate that all items had acceptable explanatory power for their corresponding constructs.

**Table 2:** Reliability and validity.

Items	Factor loadings	Cronbach's α	CR	AVE
Cognitive Reappraisal (CR)		0.898	0.898	0.594
CR1	0.767			
CR2	0.758			
CR3	0.777			
CR4	0.773			
CR5	0.778			
CR6	0.770			
Stress Self–Management (SS)		0.899	0.899	0.597
SS1	0.740			
SS2	0.766			
SS3	0.798			
SS4	0.782			
SS5	0.802			
SS6	0.743			
*HRQoL*		0.933	0.933	0.582
HRQoL1	0.775			
HRQoL2	0.745			
HRQoL3	0.779			
HRQoL4	0.745			
HRQoL5	0.734			
HRQoL6	0.768			
HRQoL7	0.756			
HRQoL8	0.779			
HRQoL9	0.771			
HRQoL10	0.775			

The internal consistency of the constructs was also satisfactory. Cronbach's alpha values were 0.898 for cognitive reappraisal, 0.899 for stress self-management, and 0.933 for HRQoL, all exceeding the recommended threshold of 0.70. The composite reliability values were also acceptable, with values of 0.898, 0.899, and 0.933, respectively. In addition, the average variance extracted values were 0.594 for cognitive reappraisal, 0.597 for stress self-management, and 0.582 for HRQoL, all above the recommended threshold of 0.50. These findings suggest that the measurement model demonstrated good reliability and convergent validity.

Discriminant validity was assessed by comparing the square root of the AVE with the correlations among the constructs. As shown in Table 3, the square roots of the AVE were 0.771 for cognitive reappraisal, 0.773 for stress self-management, and 0.763 for HRQoL. These values were greater than the corresponding inter-construct correlations, which ranged from 0.406 to 0.441. Therefore, the results provide support for the discriminant validity of the measurement model.

**Table 3:** Pearson correlation.

Construct	CR	SS	HRQoL
CR	(0.771)		
SS	0.406^**^	(0.773)	
HRQoL	0.425^**^	0.441^**^	(0.763)

The square root of the AVE is in diagonals; off diagonals are a Pearson's correlations of constructs. ^**^p < 0.01.

### 4.2 Structural model

After confirming the reliability and validity of the measurement model, structural equation modeling was conducted to test the hypothesized relationships among physical activity, cognitive reappraisal, stress self-management, and HRQoL. Gender, grade, academic stress, sleep duration, and participation in school sports clubs, sports teams, or extracurricular physical training were included as control variables. The results showed that the structural model had a good fit to the data: χ^2^/df = 1.211, GFI = 0.937, AGFI = 0.920, NFI = 0.934, IFI = 0.988, TLI = 0.986, CFI = 0.988, and RMSEA = 0.022. These fit indices indicate that the proposed model was acceptable.

**Figure 2:**
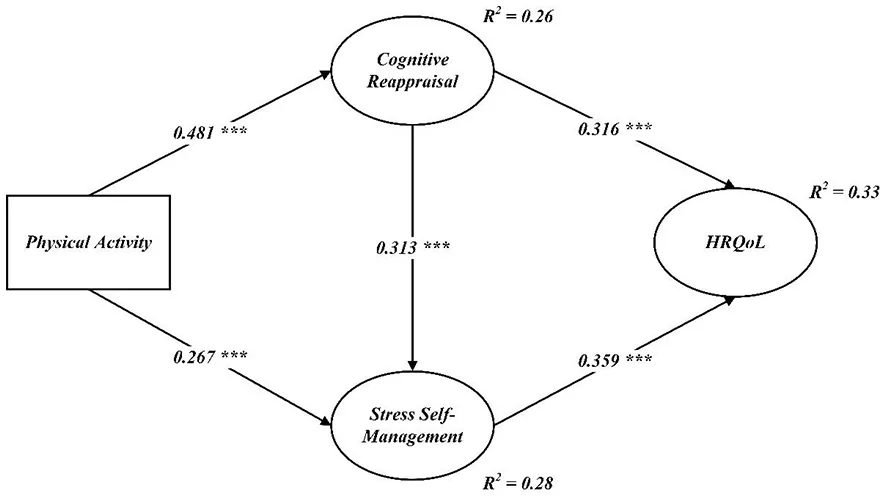
Structural model. Standardized path coefficients are reported. Gender, grade, academic stress, sleep duration, and participation in school sports clubs, sports teams, or extracurricular physical training were controlled for but are not displayed for visual clarity. ^***^p < 0.001.

After accounting for the control variables, the path analysis results continued to support the hypothesized relationships. The standardized path coefficients of the structural model are presented in Figure 2. First, physical activity was positively associated with cognitive reappraisal (β = 0.481, *p* < 0.001), supporting H1. Second, physical activity was positively associated with stress self-management (β = 0.267, *p* < 0.001), supporting H2. Third, cognitive reappraisal was positively associated with stress self-management (β = 0.313, *p* < 0.001), supporting H3. Fourth, cognitive reappraisal was positively associated with HRQoL (β = 0.316, *p* < 0.001), supporting H4. Fifth, stress self-management was positively associated with HRQoL (β = 0.359, *p* < 0.001), supporting H5.

Among the control variables, academic stress was negatively associated with cognitive reappraisal (β = −0.092, *p* = 0.044). Gender was positively associated with stress self-management (β = 0.119, *p* = 0.009), whereas participation in school sports clubs, sports teams, or extracurricular physical training was negatively associated with stress self-management (β = −0.089, *p* = 0.049). The remaining control-variable paths were not statistically significant.

The model explained 26% of the variance in cognitive reappraisal, 28% of the variance in stress self-management, and 33% of the variance in HRQoL. These results suggest that adolescents with higher levels of physical activity were more likely to report higher cognitive reappraisal and better stress self-management, which were further associated with better HRQoL, even after accounting for the selected background variables. In addition, the significant paths from physical activity to cognitive reappraisal, from cognitive reappraisal to stress self-management, and from stress self-management to HRQoL provide preliminary support for the proposed serial mediation pathway, supporting H6.

### 4.3 Mediation analysis

Mediation analysis using bootstrap estimation with 5,000 resamples and 95% bias-corrected confidence intervals indicated a significant indirect association between physical activity and HRQoL through cognitive reappraisal and stress self-management. As shown in Table 4, the standardized indirect association was 0.302 [SE = 0.034, 95% CI = (0.234, 0.371), *p* < 0.001], supporting H6.

**Table 4:** Standardized indirect effect.

Path	Point estimate	Product of coefficients	Bootstrapping		
			**Bias–corrected 95% CI**		**Two–tailed significance**
		SE	Lower	Upper	
PA → HRQoL	0.302	0.034	0.234	0.371	*p* < 0.001

## 5 Discussion

### 5.1 Theoretical contributions

This study makes several theoretical contributions to the existing literature on adolescent HRQoL, physical activity, and psychological regulation processes.

First, this study extends the existing literature on physical activity and adolescent wellbeing by moving beyond the traditional focus on direct associations. Previous research has consistently demonstrated that physical activity is positively associated with mental health outcomes and HRQoL among adolescents (10). However, much of this evidence has treated physical activity primarily as a behavioral predictor without sufficiently unpacking the psychological processes through which such effects occur. By incorporating cognitive reappraisal and stress self-management into a unified framework, this study provides a more fine-grained explanation of how physical activity is translated into improved HRQoL, thereby advancing current understanding of the behavioral–psychological linkage in adolescent health research.

Second, this study contributes to the emotion regulation literature by positioning cognitive reappraisal within a broader behavioral and coping system. Existing research on cognitive reappraisal has primarily focused on its direct effects on emotional wellbeing and psychological adjustment (52, 57). While these studies have established the importance of cognitive reappraisal as an adaptive emotion regulation strategy, less attention has been given to its behavioral antecedents and its role in shaping downstream coping processes. By linking physical activity to cognitive reappraisal and further connecting cognitive reappraisal to stress self-management, this study integrates emotion regulation theory with health behavior research, thereby extending its explanatory scope beyond intra-individual emotional processes.

Third, this study enriches the stress and coping literature by highlighting the sequential interplay between cognitive regulation and stress self-management in predicting HRQoL. Prior research has widely demonstrated that adaptive coping strategies are associated with better psychological adjustment and higher quality of life in adolescents (49, 67). However, these studies have typically examined coping processes in isolation from upstream cognitive regulation mechanisms. By empirically demonstrating that stress self-management may function as a downstream mechanism through which cognitive reappraisal operates, this study offers a more integrated view of how adolescents manage stress and maintain wellbeing.

Finally, by adopting Conservation of Resources theory as an overarching theoretical lens, this study contributes to resource-based explanations of adolescent wellbeing. Although Conservation of Resources theory has been widely applied in occupational and health psychology, its application to the integration of physical activity, emotion regulation, and stress-management processes among adolescents remains limited. The present study does not conceptualize physical activity as a resource in itself, but as a resource-investment behavior that may support the maintenance or development of personal regulatory resources. Consistent with previous applications of Conservation of Resources theory to physical activity and adolescent psychological development, the findings suggest a potential resource-gain sequence in which physical activity is associated with cognitive reappraisal and stress self-management, which are subsequently associated with higher HRQoL. This interpretation extends the application of Conservation of Resources theory while preserving the conceptual distinction between resources and behaviors through which resources may be invested, restored, or developed.

### 5.2 Practical implications

The findings of this study have several practical implications for supporting adolescents' HRQoL. First, schools should recognize physical activity not only as a means of improving physical fitness, but also as an important component of students' broader psychological development. Physical education programmes may therefore include diverse and enjoyable forms of activity, such as team sports, running, dance, yoga, and traditional activities such as tai chi. Providing students with a wider range of choices may help them develop sustainable activity habits while creating regular opportunities to disengage from academic pressure, experience positive emotions, and recover from daily stress.

Second, physical activity initiatives may be more beneficial when combined with age-appropriate support for cognitive reappraisal and stress self-management. Rather than treating physical activity, emotion regulation, and stress management as separate areas, schools could incorporate brief and practical psychological skills into physical education, health education, or pastoral programmes. For example, students could be encouraged to reconsider unhelpful interpretations of academic or interpersonal difficulties and to develop balanced routines involving exercise, rest, relaxation, and adequate sleep. School counselors may reinforce these skills through individual counseling or small-group activities, particularly for students experiencing persistent academic pressure or difficulties in managing emotions.

Third, families and mental health professionals can provide additional support according to adolescents' individual needs. Parents can encourage regular physical activity, provide opportunities for exercise, and help adolescents maintain a balanced daily schedule without presenting physical activity as competing with academic achievement. Clinical psychologists and other mental health professionals may also consider adolescents' physical activity patterns and everyday stress-management routines as part of a broader psychological assessment or intervention plan. For adolescents experiencing more substantial psychological difficulties, physical activity and stress self-management should be used as complementary elements alongside appropriate professional treatment rather than as substitutes for it.

Finally, cooperation among schools, families, communities, healthcare professionals, and policymakers is needed to create supportive environments for adolescent physical and psychological wellbeing. Communities can provide safe and accessible spaces for exercise, while schools with limited sports facilities or counseling resources may require additional institutional support. Policies that strengthen school-based physical activity, psychological education, and access to counseling services may help reduce barriers to healthy development. Overall, the findings suggest that programmes designed to promote adolescents' HRQoL should integrate accessible physical activity opportunities with the development of cognitive reappraisal and everyday stress self-management skills.

### 5.3 Limitations

Several limitations should be acknowledged. First, this study used a cross-sectional design, which limits the ability to draw causal conclusions. Although the results showed significant associations among physical activity, cognitive reappraisal, stress self-management, and HRQoL, the direction of these relationships cannot be fully confirmed. Future studies could use longitudinal or experimental designs to examine whether changes in physical activity lead to improvements in cognitive reappraisal, stress self-management, and HRQoL over time.

Second, the data were collected through self-reported questionnaires, which may introduce response bias and social desirability bias. Although the Harman's single-factor test suggested that common method variance was not a serious concern, self-report data may still be affected by students' subjective perceptions. Future research could combine self-reported data with more objective indicators, such as physical activity tracking, teacher reports, or parent reports, to improve the robustness of the findings.

Third, the participants were recruited from only two secondary schools in Jiangsu Province using convenience and snowball sampling. Therefore, the findings may not be generalizable to adolescents from other regions, school types, socioeconomic backgrounds, or cultural settings. In addition, detailed socioeconomic information about the participants was not collected, which limited our ability to examine whether the observed relationships differed across socioeconomic groups. Future studies should adopt more representative sampling strategies and include participants from diverse geographical, educational, and socioeconomic backgrounds.

## Data Availability

The raw data supporting the conclusions of this article will be made available by the authors, without undue reservation.
